# A linear-time algorithm that avoids inverses and computes Jackknife (leave-one-out) products like convolutions or other operators in commutative semigroups

**DOI:** 10.1186/s13015-020-00178-x

**Published:** 2020-09-19

**Authors:** John L. Spouge, Joseph M. Ziegelbauer, Mileidy Gonzalez

**Affiliations:** 1 National Center for Biotechnology Information, National Library of Medicine, National Institutes of Health, Room 6N603, Building 38A, Bethesda, MD 20894 USA; 2 HIV and AIDS Malignancy Branch, Center for Cancer Research, National Cancer Institute, National Institutes of Health, Bethesda, MD 20892 USA; 3 Genomics Research and Development, Lenovo HPC and AI, 1009 Think Pl, Morrisville, NC 27560 USA

**Keywords:** Commutative semigroup, Leave-one-out, Jackknife products, Segment tree, Data structure

## Abstract

**Background:**

Data about herpesvirus microRNA motifs on human circular RNAs suggested the following statistical question. Consider independent random counts, not necessarily identically distributed. Conditioned on the sum, decide whether one of the counts is unusually large. Exact computation of the p*-*value leads to a specific algorithmic problem. Given $n$ elements $g_{0} ,g_{1} , \ldots ,g_{n - 1}$ in a set $G$ with the closure and associative properties and a commutative product without inverses, compute the jackknife (leave-one-out) products $\bar{g}_{j} = g_{0} g_{1} \cdots g_{j - 1} g_{j + 1} \cdots g_{n - 1}$ ($0 \le j < n$).

**Results:**

This article gives a linear-time Jackknife Product algorithm. Its upward phase constructs a standard segment tree for computing segment products like $g_{{\left[ {i,j} \right)}} = g_{i} g_{i + 1} \cdots g_{j - 1}$; its novel downward phase mirrors the upward phase while exploiting the symmetry of $g_{j}$ and its complement $\bar{g}_{j}$. The algorithm requires storage for $2n$ elements of $G$ and only about $3n$ products. In contrast, the standard segment tree algorithms require about $n$ products for construction and $\log_{2} n$ products for calculating each $\bar{g}_{j}$, i.e., about $n\log_{2} n$ products in total; and a naïve quadratic algorithm using $n - 2$ element-by-element products to compute each $\bar{g}_{j}$ requires $n\left( {n - 2} \right)$ products.

**Conclusions:**

In the herpesvirus application, the Jackknife Product algorithm required 15 min; standard segment tree algorithms would have taken an estimated 3 h; and the quadratic algorithm, an estimated 1 month. The Jackknife Product algorithm has many possible uses in bioinformatics and statistics.

## Background

### A biological question

**Fig. 1:**
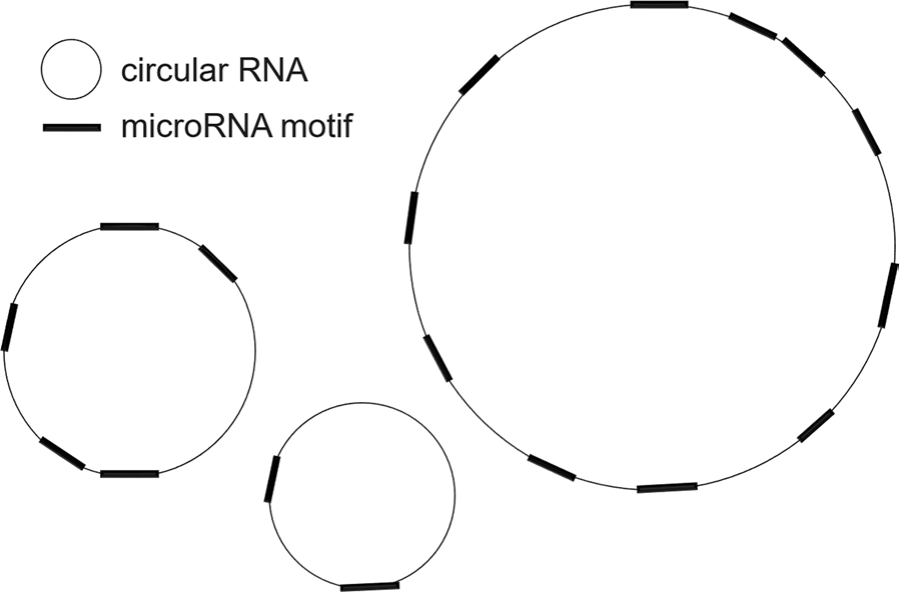
A schematic diagram of herpesvirus miRNA motif occurring on a human circRNA. As indicated in the legend, each thin circle represents a circRNA; each thick line segment, the occurrence of a miRNA motif on the corresponding circRNA. Both circRNAs and the miRNA motif have nucleotide sequences represented by IUPAC codes (A, C, G, U). This figure illustrates occurrences of a single miRNA motif (e.g., UUACAGG) on the circRNAs. The biological question is: “does any circRNA have too many occurrences of the motif to be explained by chance alone?” In the actual application, the $n = 3086$ circRNAs ranged in length from 69 nt to 158565 nt

Circular RNAs (circRNAs) are single-stranded noncoding RNAs that can inhibit another RNA molecule by binding to it, mopping it up like a sponge. During herpesvirus infection, human hosts produce circRNAs with target sites that may bind herpesvirus microRNA (miRNA) [1] (see Fig. 1). Given a sequence motif, e.g., a target site for a miRNA, researchers counted how many times the motif occurs in each circRNA sequence. They then posed a question: is the motif unusually enriched in any of the circRNAs, i.e., does any circRNA have too many occurrences of the motif to be explained by chance alone? If “yes”, the researchers could then focus their further experimental efforts on those circRNAs.

### A statistical answer

Figure 1 illustrates a set of circRNAs with varying length, with a single miRNA motif occurring as indicated on each circRNA. Let $i = 0,1 \ldots ,n - 1$ index the circRNAs; the random variate $X_{i}$ count the motif occurrences in the $i$-th circRNA; $k\left( i \right)$ equal the observed count for $X_{i}$; and the sum $S = \sum\nolimits_{i = 0}^{n - 1} {X_{i} }$ count the total motif occurrences among the circRNAs, with observed total $K = \sum\nolimits_{i = 0}^{n - 1} {k\left( i \right)}$.

The following set-up provides a general statistical test for deciding the biological question. Let $\left\{ {X_{i} :i = 0,1, \ldots ,n - 1} \right\}$ represent independent random counts (i.e., non-negative integer random variates), not necessarily identically distributed, with sum $S = \sum\nolimits_{i = 0}^{n - 1} {X_{i} }$. Given observed values $\left\{ {X_{i} = k\left( i \right):i = 0,1, \ldots ,n - 1} \right\}$ with observed sum $K = \sum\nolimits_{i = 0}^{n - 1} {k\left( i \right)}$, consider the computation of the conditional p-values ${\mathbb{P}}\left\{ {X_{i} \ge k\left( i \right)\left| {S = K} \right.} \right\}$ ($i = 0,1, \ldots ,n - 1$). The conditional p-values can decide the question: “Is any term in the sum unusually large relative to the others?”

The abstract question in the previous paragraph generalizes some common tests. For example, the standard 2 × 2 Fisher exact test [2, p. 96] answers the question in the special case of $n = 2$ categories: each $X_{i}$ has a binomial distribution with common success probability $p$, conditional on known numbers of trials $N_{i}$ ($i = 0,1$). Although the Fisher exact test generalizes directly to a single exact p-value for a $2 \times n$ table [3], the generalization can require prohibitive amounts of computation. The abstract question corresponds to a computationally cheaper alternative that also decides which columns in the $2 \times n$ table are unusual [4].

To derive an expression for the conditional p-value, therefore, let $g_{i} \left[ k \right] = {\mathbb{P}}\left\{ {X_{i} = k} \right\}$ be given, so the array $g_{i} = \left( {g_{i} \left[ 0 \right],g_{i} \left[ 1 \right], \ldots ,g_{i} \left[ K \right]} \right)$ gives the distribution of $X_{i}$, truncated at the observed total $K = \sum\nolimits_{i = 0}^{n - 1} {k\left( i \right)}$. Because $g_{i}$ is a truncated probability distribution, $g_{i} \in G$, the set of all real ($K + 1$)-tuples $\left( {g\left[ 0 \right],g\left[ 1 \right], \ldots ,g\left[ K \right]} \right)$ satisfies $g\left[ k \right] \ge 0$ ($k = 0,1, \ldots ,K$) and $\sum\nolimits_{k = 0}^{K} {g\left[ k \right]} \le 1$. The truncation still permits exact calculation of the probabilities below. To calculate the distribution of the sum $S = \sum\nolimits_{i = 0}^{n - 1} {X_{i} }$ for $S \le K$, define the truncated convolution operation $g = g^{\prime} \circ g^{\prime\prime}$, for which $g\left[ k \right] = \sum\nolimits_{j = 0}^{k} {g^{\prime}\left[ j \right]g^{\prime\prime}\left[ {k - j} \right]}$ ($k = 0,1, \ldots ,K$). Hereafter, the operation “$\circ$” is often left implicit: $g^{\prime} \circ g^{\prime\prime} = g^{\prime}g^{\prime\prime}$.

Let $\bar{g} = g_{0} g_{1} \cdots g_{n - 1}$, so $\bar{g}\left[ k \right] = {\mathbb{P}}\left\{ {S = k} \right\}$ ($k = 0,1, \ldots ,K$). Define the “jackknife products” $\bar{g}_{j} = g_{0} g_{1} \cdots g_{j - 1} g_{j + 1} \cdots g_{n - 1}$ ($0 \le j < n$) (implicitly including the products $\bar{g}_{0} = g_{1} g_{2} \cdots g_{n - 1}$ and $\bar{g}_{n - 1} = g_{0} g_{1} \cdots g_{n - 2}$). The jackknife products contain the same products as $\bar{g}$, except that in turn each skips over $g_{j}$ ($0 \le j < n$). Like the jackknife procedure in statistics, therefore, jackknife products successively omit each datum in a dataset [5].

With the jackknife products in hand, the conditional p-values are a straightforward computation:

1 $${\mathbb{P}}\left\{ {X_{i} \ge k\left( i \right)\left| {S = K} \right.} \right\} = \frac{{\sum\limits_{k = k\left( i \right)}^{K} {g_{i} \left[ k \right]\bar{g}_{i} \left[ {K - k} \right]} }}{{\bar{g}\left[ K \right]}}.$$

With respect to Eq. (1) and the biological question in Fig. 1, Appendix B gives the count $\bar{g}\left[ K \right]$ of the ways that $n$ circRNAs of known but varying length may contain $K$ miRNA motifs of equal length, the count $g_{i} \left[ k \right]$ of the ways that the $i$ th circRNA may contain $k$ motifs, and the count $\bar{g}_{i} \left[ {K - k} \right]$ of the ways that all circRNAs but the $i$ th may contain $K - k$ motifs. Appendix B derives the count $g_{i} \left[ k \right]$ for circRNAs from the easier count for placing motifs on a linear RNA molecule. For combinatorial probabilities like ${\mathbb{P}}\left\{ {X_{i} \ge k\left( i \right)\left| {S = K} \right.} \right\}$, Eq. (1) remains relevant, even if $\left\{ {g_{i} \left[ k \right]} \right\}$ are counts instead of probabilities. The biological question therefore exemplifies a commonplace computational need in applied combinatorial probability.

The Discussion indicates that in our application, transform methods can encounter substantial obstacles when computing Eq. (1) (e.g., see [6]), because the quantities in Eq. (1) can range over many orders of magnitude. This article therefore pursues direct exact calculation of ${\mathbb{P}}\left\{ {X_{i} \ge k\left( i \right)\left| {S = K} \right.} \right\}$. The product forms of $\bar{g}$ and $\left\{ {\bar{g}_{j} } \right\}$ suggest that any efficient algorithm may be abstracted to broaden its applications, as follows.

### Semigroups, groups, and commutative groups

Let $\left( {G, \circ } \right)$ denote a set $G$ with a binary product $g \circ g^{\prime}$ on its elements. Let “$g \in G$” denote “$g$ is an element of $G$”, and consider the following properties [7].

1. *Closure* $g \circ g^{\prime} \in G$ for every $g,g^{\prime} \in G$
2. *Associative* $\left( {g \circ g^{\prime}} \right) \circ g^{\prime\prime} = g \circ \left( {g^{\prime} \circ g^{\prime\prime}} \right)$ for every $g,g^{\prime},g^{\prime\prime} \in G$
3. *Identity* There exists an identity element $e \in G$, such that $e \circ g = g \circ e = g$ for every $g \in G$
4. *Commutative* $g \circ g^{\prime} = g^{\prime} \circ g$ for every $g,g^{\prime} \in G$

If the Closure and Associative properties hold, $\left( {G, \circ } \right)$ is a semigroup. Without loss of generality, we assume below that the Identity property holds. If not, adjoin an element $e \in G$, such that $e \circ g = g \circ e = g$ for every $g \in G$. In addition, if the Commutative property holds for every $g,g^{\prime} \in G$, the semigroup $\left( {G, \circ } \right)$ is commutative. Unless stated otherwise hereafter, $\left( {G, \circ } \right)$ denotes a commutative semigroup. The Jackknife Product algorithm central to this article is correct in a commutative semigroup.

(5) *Inverse* For every $g \in G$, there exists an inverse $g^{ - 1} \in G$, such that $g \circ g^{ - 1} = g^{ - 1} \circ g = e$

As shown later, the Jackknife Product algorithm does not require the Inverse property. In passing, note that the convolution semigroup relevant to the circRNA–miRNA application lacks the Inverse property, as does any convolution semigroup for calculating p-values, e.g., the ones relevant to sequence motif matching [6]. To demonstrate, let $X,Y \ge 0$ be independent integer random variates. The identity $e$ for convolution corresponds to the variate $Z = 0$, because $0 + X = X + 0 = X$ for every variate $X$. If $X + Y = 0$, however, the independence of $X$ and $Y$ implies that both are constant and therefore $X = Y = 0$. In the relevant convolution semigroup, therefore, all elements except the identity $e$ lack an inverse.

The non-zero real numbers under ordinary multiplication form a commutative semigroup $\left( {G, \circ } \right)$ with the Inverse property. They provide a familiar setting for discussing some algorithmic issues when computing $\left\{ {\bar{g}_{j} } \right\}$. Let $\bar{g} = g_{0} g_{1} \cdots g_{n - 1}$ be the usual product of $n$ real numbers, and consider the toy problem of computing all jackknife products $\left\{ {\bar{g}_{j} } \right\}$ that omit a single factor $g_{j}$ ($0 \le j < n$) from $\bar{g}$. Inverses $\left\{ {g_{j}^{ - 1} } \right\}$ are available, so an obvious algorithm computes $\bar{g}$ and then $\left\{ {\bar{g}_{j} = \bar{g}g_{j}^{ - 1} } \right\}$ with $n$ inverses and $2n - 1 = \left( {n - 1} \right) + n$ products. If the inverses were unavailable, however, the naïve algorithm using $n - 2$ element-by-element products to compute each $\left\{ {\bar{g}_{j} } \right\}$ would require $n\left( {n - 2} \right)$ products. The quadratic time renders the naïve algorithm impractical for many applications.

Figure 1 illustrates a standard data structure called a segment tree, omitting the root at the top of the segment tree. Algorithms based solely on a segment tree can calculate the jackknife products $\left\{ {\bar{g}_{j} } \right\}$ in time $O\left( {n\log n} \right)$, fast enough for many applications. The segment tree computes segment products like $g_{{\left[ {i,j} \right)}} = g_{i} g_{i + 1} \cdots g_{j - 1}$ without using the commutative property, so it can similarly compute jackknife products like $\left\{ {\bar{g}_{j} = g_{{\left[ {0,j} \right)}} g_{{\left[ {j + 1,n} \right)}} } \right\}$. If the semigroup $\left( {G, \circ } \right)$ is commutative, however, a Jackknife Product algorithm can avoid inverses and reduce the computational time further, from $O\left( {n\log n} \right)$ to $O\left( n \right)$. With in-place computations requiring only the space for the segment tree, the Jackknife Product algorithm avoids inverses yet still requires only about $3n$ products and storage for $2n$ numbers. It is therefore surprisingly economical, even when compared to the obvious algorithm using inverses. Indeed, our application to circular RNA required some economy, with its convolution of $n = 3086$ distributions, some truncated only after $K = 997$ terms. In a general statistical setting, convolutions form a commutative semigroup $\left( {G, \circ } \right)$ without inverses, so our application already indicates that the Jackknife Product algorithm has broad applicability.

## Theory

Appendix A proves the correctness of the Jackknife Product algorithm given below.

### The Jackknife Product algorithm

**Fig. 2:**
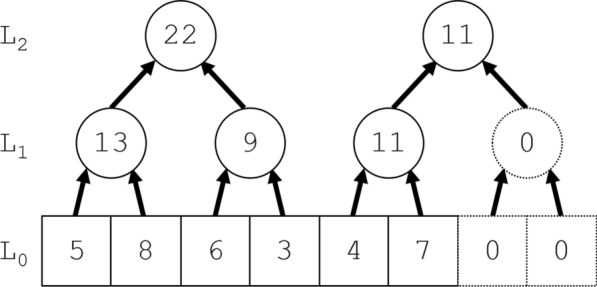
A (rootless) segment tree. This figure illustrates the rootless segment tree constructed in the upward phase of the Jackknife Product algorithm. The commutative semigroup $\left( {G, \circ } \right)$ illustrated is the set of nonnegative integers under addition. The bottom row of $n^{*} = 2^{m}$ squares ($m = 3$) contains $L_{0} \left[ j \right] = g_{j}$ ($0 \le j < n^{*}$). In the next row up, as indicated by the arrow pairs leading into each circle, the array $L_{1}$ contains consecutive sums of consecutive disjoint pairs in $L_{0}$, e.g., $L_{1} \left[ 0 \right] = 13 = 5 + 8$. The rest of the segment tree is constructed recursively upward to $L_{m - 1}$, just as $L_{1}$ was constructed from $L_{0}$. Here, 2 copies of the additive identity $e = 0$ pad out $L_{0}$ on the right. Padded on the right, the copies contribute literally nothing to the segment tree above them. Their non-contributions have dotted outlines

Let $\left( {G, \circ } \right)$ be a commutative semigroup. The Jackknife Product algorithm has three phases: upward, downward, and transposition. Its upward phase simply constructs a segment tree (see Fig. 1); its downward phase exploits the symmetry of $g_{j}$ and its complement $\bar{g}_{j}$ to mirror the upward phase while computing $\left\{ {\bar{g}_{j} } \right\}$ (see Fig. 2); and its final transposition phase then swaps successive pairs in an array (not pictured). As Figs. 1 and 2 suggest, the three phases yield a simpler algorithm if $n = n^{ * } = 2^{m}$ is a binary power. To recover the $n^{ * } = 2^{m}$ algorithm from them, pad $\left\{ {g_{j} } \right\}$ on the right with copies of the identity $e$ up to $n^{ * }$ elements, where $n^{ * } = 2^{m}$ is the smallest binary power greater than or equal to $n$, i.e., replace $\left\{ {g_{j} } \right\}$ with $\left\{ {g_{0} ,g_{1} \ldots ,g_{n - 1} ,e, \ldots ,e} \right\}$, with $n^{ * } - n$ copies of $e$. The $n^{ * } = 2^{m}$ algorithm can therefore pad any input of $n$ elements up to $n^{ * } = 2^{m}$ elements without loss of generality. The algorithm given below is therefore slightly more intricate than the $n^{ * } = 2^{m}$ algorithm, but it may save almost a factor of 2 in storage and time by omitting the padded copies of $e$. In any case, the simpler algorithm can always be recovered from the phases for general $n$ given here, if desired.

**Fig. 3:**
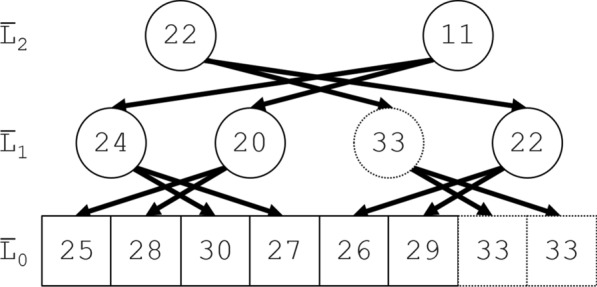
A (rootless) complementary segment tree. This figure illustrates the rootless complementary segment tree constructed in the downward phase of the Jackknife Product algorithm from the rootless segment tree in Fig. 2. The downward phase starts by initializing the topmost row $\bar{L}_{m - 1}$ ($m = 3$) with the topmost row $L_{m - 1}$ of the rootless segment tree. The row $L_{2}$ in Fig. 2 and the row $\bar{L}_{2}$ in Fig. 3, e.g., contain 22 and 11. For each $\bar{L}_{k - 1} \left[ j \right]$ in Fig. 3, downward arrows run from $\bar{L}_{k} \left[ {\alpha_{k} \left( j \right)} \right]$ to $\bar{L}_{k - 1} \left[ j \right]$. As they indicate, each node in $\bar{L}_{k}$ contributes to its 2 “nieces” in Fig. 2 to produce the next row down in Fig. 3, e.g., $\bar{L}_{2} \left[ 1 \right] = 11$ contributes to its nieces $L_{1} \left[ 0 \right] = 13$ and $L_{1} \left[ 1 \right] = 9$ in the segment, to produce $\bar{L}_{1} \left[ 0 \right] = 13 + 11 = 24$ and $\bar{L}_{1} \left[ 1 \right] = 9 + 11 = 20$ in the complementary segment tree. The rest of the complementary segment tree is constructed recursively downward to $\bar{L}_{0}$, just as $\bar{L}_{1}$ was constructed from $\bar{L}_{2}$. In Fig. 2, the elements of $L_{0}$ (in squares) total 33. To demonstrate the effect of the Jackknife Product algorithm, subtract in turn in Fig. 3 each element (25, 28, 30, 27, 26, 29, 33, 33) in the bottom row $\bar{L}_{0}$ from the total 33. The result (8, 5, 3, 6, 7, 4, 0, 0) is the bottom row $L_{0}$ in Fig. 2 with successive pairs transposed, so $\bar{L}_{0} \left[ j \right] = \bar{g}_{\tau \left( j \right)}$, or equivalently $\bar{g}_{j} = \bar{L}_{0} \left[ {\tau \left( j \right)} \right]$

We start with notational preliminaries. Define the floor function $\left\lfloor x \right\rfloor = \hbox{max} \left\{ {j:j \le x} \right\}$ and the ceiling function $\left\lceil x \right\rceil = \hbox{min} \left\{ {j:x \le j} \right\}$ (both standard); and the binary right-shift function $\rho \left( j \right) = \left\lfloor {j/2} \right\rfloor$. Other quantities also smooth our presentation. Given a product $\bar{g} = g_{0} g_{1} \cdots g_{n - 1}$ of interest, define $m = \left\lceil {\log_{2} n} \right\rceil$ and $n_{k} = \left\lceil {n2^{ - k} } \right\rceil$ for $0 \le k < m$. Below, the symbol “□” connotes the end of a proof.

### The upward phase

The upward phase starts with the initial array $L_{0} \left[ j \right] = g_{j}$ ($0 \le j < n$) and simply computes a standard (but rootless) segment tree consisting of segment products $L_{k} \left[ j \right]$ for $j = 0,1, \ldots ,n_{k} - 1$ and $k = 0,1, \ldots ,m - 1$.
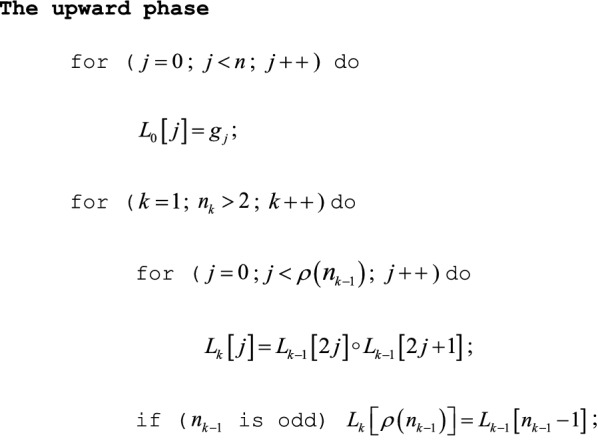


### Comments

(1) If $n = n^{ * } = 2^{m}$ is a binary power, $\rho \left( {n_{k - 1} } \right) = n_{k} = 2^{m - k}$ and the final line in the upward phase can be omitted. (2) Of some peripheral interest, Laaksonen [8] gives the algorithm in a different context, embedding a binary tree in a single array of length $O\left( n \right)$. If any $L_{0} \left[ j \right] = g_{j}$ changes, he also shows how to update the single array with $O\left( {\log n} \right)$ multiplications. If the downward phase (next) does not overwrite the segment tree $\left\{ {L_{k} } \right\}$ by using in-place computation, it permits a similar update.

### The downward phase

The transposition function $\tau \left( j \right) = j + \left( { - 1} \right)^{j}$ transposes adjacent indices, e.g., $\left( {L_{\tau \left( 0 \right)} ,L_{\tau \left( 1 \right)} ,L_{\tau \left( 2 \right)} ,L_{\tau \left( 3 \right)} } \right) = \left( {L_{1} ,L_{0} ,L_{3} ,L_{2} } \right)$. We also require $\alpha_{k} \left( j \right) = \hbox{min} \left\{ {\tau \rho \left( j \right),n_{k} - 1} \right\}$ for $0 \le j < n_{k - 1}$ and $1 \le k < m$, the index of “aunts”, as illustrated by Fig. 2. Just as Fig. 1 illustrates a rootless segment tree in the upward phase, Fig. 2 illustrates the corresponding rootless complementary segment tree in the downward phase.

The downward phase computes complementary segment products $\bar{L}_{k} \left[ j \right]$ for $j = 0,1 \ldots ,n_{k} - 1$ and $k = m - 1,m - 2, \ldots ,0$.
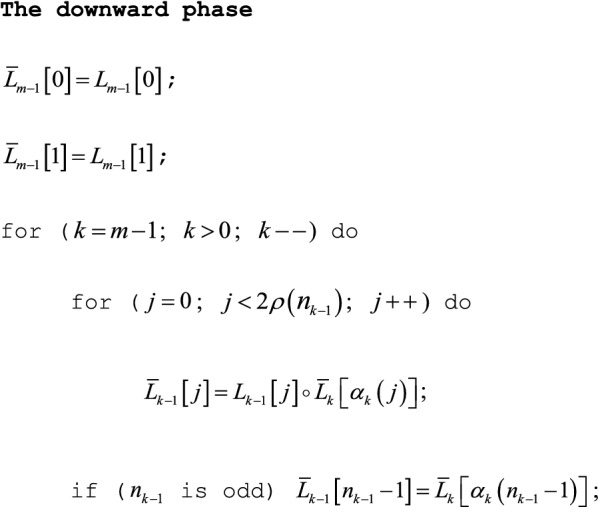


### Comments

(1) If $n = n^{ * } = 2^{m}$ is a binary power, $\rho \left( {n_{k - 1} } \right) = n_{k} = 2^{m - k}$, $\alpha_{k} \left( j \right) = \tau \rho \left( j \right)$, and the final line in the downward phase can be omitted. (2) The downward phase can be modified in the obvious fashion to permit in-place calculation of $\bar{L}_{k - 1} \left[ j \right]$ from $L_{k - 1} \left[ j \right]$, reducing total memory allocation by about 2.

As Appendix A proves, the final array $\bar{L}_{0}$ has elements $\bar{L}_{0} \left[ {\tau \left( j \right)} \right] = \bar{g}_{j}$ ($0 \le j < 2\rho \left( n \right)$), with an additional final element $\bar{L}_{0} \left[ {n - 1} \right] = \bar{g}_{n - 1}$ if $n_{0} = n$ is odd, so the Jackknife Product algorithm ends with a straightforward transposition phase.
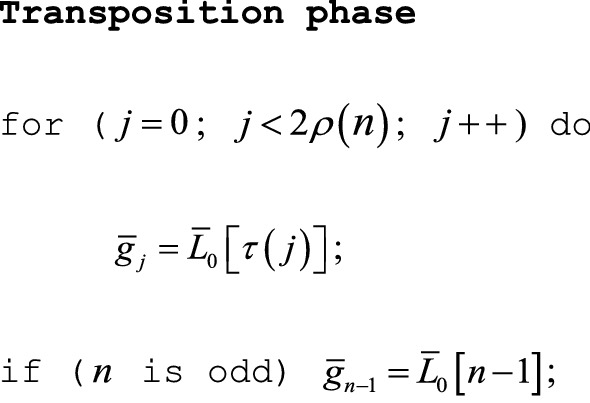


### Comments

The transposition phase can permit an in-place calculation of $\left\{ {\bar{g}_{j} } \right\}$ to overwrite $\bar{L}_{0}$.

### Computational time and storage

Note $n_{j} = \left\lceil {n2^{ - j} } \right\rceil$, so $0 \le n_{j} - n2^{ - j} < 1$. To compute $L_{k}$ from $L_{k - 1}$ or to compute $\bar{L}_{k}$ from $L_{k}$ and $\bar{L}_{k + 1}$, the Jackknife Product algorithm requires $n_{k}$ products. For large $n$, therefore, the upward phase computing the segment tree requires about $\sum\nolimits_{j = 1}^{m - 1} {n_{j} } \approx \sum\nolimits_{j = 1}^{\infty } {n2^{ - j} } = n$ products; the downward phase, about $\sum\nolimits_{j = 0}^{m - 2} {n_{j} } \approx 2n$ products. Likewise, if the downward and transposition phases compute in place by replacing $L_{k}$ with $\bar{L}_{k}$ and $\bar{L}_{0}$ with $\left\{ {\bar{g}_{j} } \right\}$, the algorithm storage is $\sum\nolimits_{j = 0}^{m - 1} {n_{j} } \approx 2n$ semigroup elements. Each of the three estimates just given for products and storage have an error bounded by $m = \left\lceil {\log_{2} n} \right\rceil$. Although the case of general $n$ could be handled by the algorithm for binary powers $n^{ * } = 2^{m}$ by setting $m = \left\lceil {\log_{2} n} \right\rceil$ and $g_{n} = g_{n + 1} = \ldots = g_{{n^{ * } - 1}} = e$, the truncated arrays in the Jackknife Product algorithm for general $n$ save about a factor of $1 \le n^{ * } /n < 2$ in both products and storage.

As written, the conditional copy statements at the end of the upward and downward phases replicate elements already in storage. If the downward phase of the Jackknife Product algorithm is implemented with in-place computation of $\bar{L}_{k - 1} \left[ j \right]$ from $L_{k - 1} \left[ j \right]$, the copy statements ensure that the algorithm never overwrites any array element it needs later. Some statements may copy some elements more than once (and therefore unnecessarily), but a negligible $m = \left\lceil {\log_{2} n} \right\rceil$ copies at most are unnecessary.

The complementary segment tree in Fig. 2 implicitly indicates the nodes in the segment tree required to compute $L_{0} \left[ {\tau \left( j \right)} \right] = \bar{g}_{j}$ for each $\bar{g}_{j}$, i.e., exactly one node in each row $L_{k}$ ($k = 0,1, \ldots ,m - 1$). Alone, the segment tree therefore requires at least $n\log_{2} n$ multiplications to compute $\left\{ {\bar{g}_{j} } \right\}$.

## Results

Appendix B gives the combinatorics relevant to the circRNA-miRNA application described in “Background” section. As is typical in combinatorial probability, the quantities $\left\{ {g_{i} \left[ k \right]} \right\}$ were counts of configurations, here, the ways of placing miRNA motifs on circRNAs. The length of each motif was *m *= 7; the largest circRNA (hsa-circ-0003473) contained $I$ = 158,565 nt, and the most abundant motif (CCCAGCU, for the m12-9star miRNA family) appeared $K$ = 997 times, so the $\left\{ {g_{i} \left[ k \right]} \right\}$ spanned thousands of orders of magnitude in Eq. (13) of Appendix B, from $g_{i} \left[ 0 \right] = 1$ to $g_{I} \left[ K \right] \approx 10^{2608}$. In Eq. (1), the dimension $K$ controls the number of terms in the convolutions. In the application, over each miRNA motif examined, the maximum number of motif occurrences on the circRNAs was $K = 997$. An Intel Core i7-3770 CPU computed the p-value relevant to the biological application on June 17, 2015. To compare later with estimated times for competing algorithms, the Jackknife Product algorithm with $n = 3086$ computed the relevant p-values in about 45 min, requiring about $3n$ products. In the application, therefore, $n$ products required about 15 min.

The application of this article to circRNA–miRNA data appears elsewhere [1].

## Discussion

This article has presented a Jackknife Product algorithm, which applies to any commutative semi-group $\left( {G, \circ } \right)$. The biological application to a circRNA–miRNA system exemplifies a general statistical method in combinatorial probability. In turn, the application in combinatorial probability exemplifies an even more general statistical test for whether a term in a sum of independent counting variates (not necessarily identically distributed) is unusually large.

Many biological contexts lead naturally to sums of independent counting variates. Domain alignments of proteins from cancer patients, e.g., display point mutations in their columns. For a given domain, a column with an excess of mutations might be inferred to cause cancer [9]. The Background section gives the pattern: let $X_{i}$ represent the mutation count in column $i = 0,1, \ldots ,n - 1$, with total mutations $S = \sum\nolimits_{i = 0}^{n - 1} {X_{i} }$. Given observed mutation counts $\left\{ {X_{i} = k\left( i \right):i = 0,1, \ldots ,n - 1} \right\}$ with observed sum $K = \sum\nolimits_{i = 0}^{n - 1} {k\left( i \right)}$, the conditional p-values ${\mathbb{P}}\left\{ {X_{i} \ge k\left( i \right)\left| {S = K} \right.} \right\}$ ($i = 0,1, \ldots ,n - 1$) can decide the question: “Does any column have an excess of mutations?” The actual application used other, very different statistical methods [9]. Unlike those methods, however, our methods can incorporate information from control (non-cancer) protein sequences to set column-specific background distributions for $\left\{ {X_{i} } \right\}$.

The Benjamini–Hochberg procedure for controlling the false discovery rate in multiple tests requires either independent p-values [10] or dependent p-values with a positive regression dependency property [11]. Loosely, the positive regression dependency property means that the p-values tend to be small together, i.e., under the null hypothesis, given that one p-value is small, then the other p-values tend to be smaller also. Inconveniently, our null hypothesis posits a fixed sum of independent counting variates, so if one variate is large and has a small p-value, it tends to reduce the other variates and increase their p-values. The circRNA-miRNA application therefore violates the statistical hypotheses of the Benjamini–Hochberg procedure. Fortunately, in the circRNA-miRNA application, a Bonferroni multiple test correction [12] sufficed because empirically, any p-value was either close to 1 or extremely small.

The Results state that for $n = 3086$, the Jackknifed Product algorithm computed the relevant p-values in about 45 min, with $n$ products requiring about 15 min of computation. In contrast, the naïve algorithm avoiding inverses and requiring $n\left( {n - 2} \right)$ products would have taken about $3086*15$ min, i.e., about 1 month. As explained under the “Computational Time and Storage” heading in the Theory section, without exploiting the special form of the jackknife products $\left\{ {\bar{g}_{j} } \right\}$, a segment tree requires about $n$ products for its construction and at least $n\log_{2} n$ products for the computation of the products $\left\{ {\bar{g}_{j} } \right\}$. Alone, segment tree algorithms would therefore have taken a minimum of about $\left( {1 + \log_{2} 3086} \right)*15$ min, i.e., about 3 h.

The convolutions in Eq. (1) might suggest that jackknife products are susceptible to computation with Fourier or Laplace transforms, which convert convolutions into products. “Results” section notes that in the biological application, however, $\left\{ {g_{i} \left[ k \right]} \right\}$ in Eq. (1) spanned thousands of orders of magnitude, at least from $g_{i} \left[ 0 \right] = 1$ to $g_{I} \left[ K \right] \approx 10^{2608}$, obstructing the direct use of transforms (e.g., see [6]). On one hand, the widely varying magnitudes necessitated an internal logarithmic representation of $\left\{ {g_{i} \left[ k \right]} \right\}$ in the computer, a minor inconvenience for direct computation with the Jackknife Product algorithm. On the other hand, they might have presented a substantial obstacle for transforms. The famous Feynman anecdote about Paul Olum’s $\tan \left( {10^{100} } \right)$ problem indicates the reason [13]:

*So Paul is walking past the lunch place and these guys are all excited. “Hey, Paul!” they call out. “Feynman’s terrific! We give him a problem that can be stated in ten seconds, and in a minute he gets the answer to 10 percent. Why don’t you give him one?” Without hardly stopping, he says, “The tangent of 10 to the 100th.” I was sunk: you have to divide by pi to 100 decimal places! It was hopeless.*

The Jackknife Product algorithm also abstracts to any commutative semigroup $\left( {G, \circ } \right)$, broadening its applicability enormously. As usual, abstraction eases debugging. Consider, e.g., the commutative semigroup consisting of all bit strings of length $n$ under the bitwise “or” operation. If the bit string $g_{j}$ has 1 in the *j*-th position and 0 s elsewhere, then the segment product $g_{{\left[ {i,j} \right)}}$ equals the bit string with 1 s in positions $\left[ {i,j} \right) = \left\{ {i,i + 1, \ldots ,j - 1} \right\}$ and 0 s elsewhere. Similarly, the complementary segment product $g_{{\overline{{\left[ {i,j} \right)}} }} = g_{{\left[ {0,i} \right)}} g_{{\left[ {j,n} \right)}}$ equals the bit string with 0 s in positions $\left[ {i,j} \right) = \left\{ {i,i + 1, \ldots ,j - 1} \right\}$ and 1 s elsewhere. The Jackknife Product algorithm is easily debugged with output consisting of the segment and complementary segment trees for the bit strings.

As a final note, even if a semigroup $\left( {G, \circ } \right)$ lacks the Commutative property, the general product algorithm for a segment tree can still compute $\left\{ {\bar{g}_{j} = g_{{\left[ {0,j} \right)}} g_{{\left[ {j + 1,n} \right)}} } \right\}$ in time $O\left( {n\log n} \right)$. In a commutative semigroup $\left( {G, \circ } \right)$, however, the downward phase of the Jackknife Product algorithm exploits the special form of the products $\left\{ {\bar{g}_{j} } \right\}$ to decrease the time to $O\left( n \right)$.

## Conclusions

This article has presented a Jackknife Product algorithm, which applies to any commutative semi-group $\left( {G, \circ } \right)$. The biological application to a circRNA–miRNA system uses a commutative semigroup of truncated convolutions to exemplify a specific application to combinatorial probabilities. In turn, the specific application in combinatorial probability exemplifies an even more general statistical test for whether a term in a sum of independent counting variates (not necessarily identically distributed) is unusually large. The general statistical test can evaluate the results of searching for a sequence or structure motif, or several motifs simultaneously. As “Discussion” section explains, the test violates the hypotheses of the Benjamini–Hochberg procedure for estimating false discovery rates, but fortunately the Bonferroni and other multiple-test corrections remain available to control familywise errors. Abstraction from convolutions to commutative semi-groups broadens the algorithm’s applicability even further. If an application only requires jackknife products $\left\{ {\bar{g}_{j} } \right\}$ and their number $n$ is large enough, “Results” and “Theory” sections show that the linear time of the Jackknife Product algorithm can make it well worth the programming effort.

## Data Availability

A self-testing, annotated file “jls_jackknifeproduct.py” implementing an in-place Jackknife Product algorithm in Python is available without any restrictions at https://github.com/johnlspouge/jackknife-product. Data were previously published elsewhere [1].

## Appendix A

This appendix proves the correctness of the Jackknife Product algorithm in “Theory” section.

Let $L_{k}$ have length $l_{k}$; $\bar{L}_{k}$, length $\bar{l}_{k}$. Some observations about $l_{k}$, $\bar{l}_{k}$, and $n_{k}$ facilitate later analysis. Because $\left\lceil {\left\lceil x \right\rceil /2} \right\rceil = \left\lceil {x/2} \right\rceil$, $\left\{ {n_{k} } \right\}$ satisfies the recursion $n_{k} = \left\lceil {n2^{ - k} } \right\rceil = \left\lceil {n2^{{ - \left( {k - 1} \right)}} /2} \right\rceil = \left\lceil {n_{k - 1} /2} \right\rceil$, with initial value $n_{0} = n$ and final value $n_{m - 1} = 2$.

### **Proposition 1**

$$l_{k} = \bar{l}_{k} = n_{k}$$ for $$0 \le k < m$$.

### Proof (by induction)

$l_{0} = n = n_{0}$. If $l_{k - 1} = n_{k - 1}$ for any $1 \le k < m$, the upward phase of the Jackknife Product algorithm shows: (1) if $n_{k - 1}$ is even, $l_{k} = \rho \left( {n_{k - 1} } \right)$; and (2) if $n_{k - 1}$ is odd, $l_{k} = \rho \left( {n_{k - 1} } \right) + 1$. In either case, $l_{k} = \left\lceil {n_{k - 1} /2} \right\rceil = n_{k}$. Thus, $l_{k} = n_{k}$ for $0 \le k < m$.

Similarly, the downward phase shows: (1) if $n_{k - 1}$ is even, $\bar{l}_{k - 1} = 2\rho \left( {n_{k - 1} } \right) = n_{k - 1}$; if $n_{k - 1}$ is odd, $\bar{l}_{k - 1} = 2\rho \left( {n_{k - 1} } \right) + 1 = n_{k - 1}$. It therefore initializes $\bar{L}_{m - 1}$ with $\bar{l}_{m - 1} = n_{m - 1} = 2$ elements and assigns $\bar{l}_{k - 1} = n_{k - 1}$ ($1 \le k < m$) elements to $\bar{L}_{k - 1}$. □

Proposition 1 and its proof ensure that with the possible exception of $\alpha_{k} \left( j \right)$ in the downward phase, all array indices in the Jackknife Product algorithm lie within the array bounds of $L_{k}$ and $\bar{L}_{k}$. Moreover, case-by-case analysis of the definition of $\alpha_{k}$ shows that $\alpha_{k} \left( j \right)$ ($0 \le j < n_{k - 1}$) always falls within the array bounds $0 \le \alpha_{k} \left( j \right) < n_{k}$ of $\bar{L}_{k}$. Inspection of the upward and downward phases shows that they define every array element before using it. With array bounds and definitions in hand, to verify the Jackknife Product algorithm, it therefore suffices to check conditions satisfied by individual elements of $L_{k}$ and $\bar{L}_{k}$. We examine first the case of binary powers $n = n^{ * } = 2^{m}$, and afterwards the case of general $n$.

*Proof of Correctness for Binary Powers* $n^{ * } = 2^{m} \ge 2$

In this subsection, some entities pertaining to binary powers $n^{ * }$ receive stars (e.g., $n^{ * }$, $n_{k}^{ * }$, $L_{k}^{ * }$, $\bar{L}_{k}^{ * }$), to distinguish them later from the corresponding entities for general $n$.

For convenience in Appendix A only, drop “$g$” in the notation $g_{{\left[ {i,j} \right)}}$, and abbreviate the segment product $g_{{\left[ {i,j} \right)}} = g_{i} g_{i + 1} \cdots g_{j - 1}$ by the corresponding half-open interval $\left[ {i,j} \right)$ and the complementary segment product $g_{0} g_{1} \cdots g_{i - 1} g_{j} \cdots g_{{n^{ * } - 1}}$ by $\overline{{\left[ {i,j} \right)}}$. The notation provides a mnemonic aid, used without comment below: for $i < j < k$, $\left[ {i,j} \right) \circ \left[ {j,k} \right) = \left[ {i,k} \right)$, $\left[ {i,j} \right) \circ \overline{{\left[ {i,k} \right)}} = \overline{{\left[ {j,k} \right)}}$ and $\left[ {j,k} \right) \circ \overline{{\left[ {i,k} \right)}} = \overline{{\left[ {i,j} \right)}}$, i.e., the product $\circ$ on sub-products behaves like a set-theoretic union of the corresponding intervals, and complementary sub-products behave like the corresponding set-theoretic complements of intervals.

The Commutative property is required to justify the correspondence between set-theoretic operations and products, e.g., the equality $\left[ {i,j} \right) \circ \overline{{\left[ {i,k} \right)}} = \overline{{\left[ {j,k} \right)}}$ commutes the segment products: examine, e.g., the second equality in the equation

2 $$\begin{aligned} g_{{\left[ {i,j} \right)}} \circ \left( {g_{{\left[ {0,i} \right)}} \circ g_{{\left[ {k,n*} \right)}} } \right) &= \left( g_{{\left[ {i,j} \right)}} \circ {g_{{\left[ {0,i} \right)}} } \right) \circ g_{{\left[ {k,n*} \right)}} \\ &= \left( {g_{{\left[ {0,i} \right)}} \circ g_{{\left[ {i,j} \right)}} } \right) \circ g_{{\left[ {k,n*} \right)}} = g_{{\left[ {0,j} \right)}} \circ g_{{\left[ {k,n*} \right)}} .\end{aligned}$$

#### **Proposition 2**

For $$0 \le k < m$$, $$L_{k}^{ * } \left[ j \right] = \left[ {j \times 2^{k} ,\left( {j + 1} \right) \times 2^{k} } \right)$$ ($$0 \le j < n_{k}^{*}$$).

#### *Proof*

See Fig. 1. The array $L_{0}^{ * }$ at generation $k = 0$ initializes the upward phase, where

3 $$L_{0}^{ * } \left[ j \right] = g_{j} = \left[ {j \times 2^{0} ,\left( {j + 1} \right) \times 2^{0} } \right)\quad {\text{for}}\quad 0 \le j < n^{ * } .$$

Thus, Proposition 2 is true for $k = 0$. Given the array $L_{k - 1}^{ * }$ at generation $k - 1$ in the upward phase, the array $L_{k}^{ * }$ at level $k$ contains products of successive adjacent pairs of elements in $L_{k - 1}^{ * }$:

4 $$\begin{aligned} L_{k}^{ * } \left[ j \right] & = L_{k - 1}^{ * } \left[ {2j} \right] \circ L_{k - 1}^{ * } \left[ {2j + 1} \right] \\ & = \left[ {2j \times 2^{k - 1} ,\left( {2j + 1} \right) \times 2^{k - 1} } \right) \circ \left[ {\left( {2j + 1} \right) \times 2^{k - 1} ,\left( {2j + 2} \right) \times 2^{k - 1} } \right) \\ & = \left[ {j \times 2^{k} ,\left( {j + 1} \right) \times 2^{k} } \right) \\ \end{aligned}$$

for $0 \le j < n_{k}^{ * } = n_{k - 1}^{ * } /2$. The upward phase terminates with $L_{m - 1}^{ * } = \left( {\left[ {0,2^{m - 1} } \right),\left[ {2^{m - 1} ,2^{m} } \right)} \right)$, so Proposition 2 is true for $0 \le k < m$. □

#### **Proposition 3**

For $$0 \le k < m$$, $$\bar{L}_{k}^{ * } \left[ j \right] = \overline{{\left[ {\tau \left( j \right) \times 2^{k} ,\left( {\tau \left( j \right) + 1} \right) \times 2^{k} } \right)}}$$ ($$0 \le j < n_{k}^{ * }$$).

### Comment

Propositions 3 and 2 formalize the previously mentioned complementary symmetry between the upward and downward phases. Because $\tau \left( {\tau \left( j \right)} \right) = j$ (i.e., transposition is idempotent), $\bar{L}_{0}^{ * } \left[ {\tau \left( j \right)} \right] = \overline{{\left[ {j,j + 1} \right)}} = \bar{g}_{j}$ for $0 \le j < n_{0}^{ * } = n^{ * }$. Thus, $\bar{L}_{0}^{ * }$ contains all jackknife products.

#### *Proof*

See Fig. 2. For $0 \le j < n_{m - 1}^{ * } = 2$, the first two lines of pseudo-code in the downward phase and Proposition 2 for $k = m - 1$ show that for $j \in \left\{ {0,1} \right\}$,

5 $$\bar{L}_{m - 1}^{ * } \left[ j \right] = L_{m - 1}^{ * } \left[ j \right] = \left[ {j \times 2^{m - 1} ,\left( {j + 1} \right) \times 2^{m - 1} } \right) = \overline{{\left[ {\tau \left( j \right) \times 2^{m - 1} ,\left( {\tau \left( j \right) + 1} \right) \times 2^{m - 1} } \right)}} ,$$

so Proposition 3 holds for $k = m - 1$. We proceed by descending induction on $k$.

For even $j$ on one hand, $2\rho \left( j \right) = j < j + 1 = \tau \left( j \right) < \tau \left( j \right) + 1 = 2\left( {\rho \left( j \right) + 1} \right)$. For $0 \le j < n_{k - 1}^{ * }$, therefore,

6 $$\left[ {j \times 2^{k - 1} ,\left( {j + 1} \right) \times 2^{k - 1} } \right) \circ \left[ {\tau \left( j \right) \times 2^{k - 1} ,\left( {\tau \left( j \right) + 1} \right) \times 2^{k - 1} } \right) = \left[ {\rho \left( j \right) \times 2^{k} ,\left( {\rho \left( j \right) + 1} \right) \times 2^{k} } \right) .$$

For odd $j$ on the other hand, $2\rho \left( j \right) = \tau \left( j \right) < \tau \left( j \right) + 1 = j < j + 1 = 2\left( {\rho \left( j \right) + 1} \right)$, so Eq. (6) holds with the factors on the left reversed, an irrelevant difference in a commutative semigroup $\left( {G, \circ } \right)$.

For $0 \le j < n_{k - 1}^{ * }$, then, if $1 \le k < m$, Proposition 2 for $k - 1$ and Proposition 3 for $k$ yield

7 $$\begin{aligned} \bar{L}_{k - 1}^{ * } \left[ j \right] & = L_{k - 1}^{ * } \left[ j \right] \circ \bar{L}_{k}^{ * } \left[ {\tau \rho \left( j \right)} \right] \\ & = \left[ {j \times 2^{k - 1} ,\left( {j + 1} \right) \times 2^{k - 1} } \right) \circ \overline{{\left[ {\rho \left( j \right) \times 2^{k} ,\left( {\rho \left( j \right) + 1} \right) \times 2^{k} } \right)}} \\ & = \overline{{\left[ {\tau \left( j \right) \times 2^{k - 1} ,\left( {\tau \left( j \right) + 1} \right) \times 2^{k - 1} } \right)}} . \\ \end{aligned}$$

Thus, Proposition 3 for $k$ implies Proposition 3 for $k - 1$ ($1 \le k < m$). □

The Jackknife Product algorithm therefore computes $\bar{L}_{0}^{ * } \left[ {\tau \left( j \right)} \right] = \overline{{\left[ {j,j + 1} \right)}} = \bar{g}_{j}$, as desired.

*Proof of correctness for general* $n \ge 2$: For general $n \ge 2$, initialize the upward phase with $L_{0} = \left( {g_{0} ,g_{1} , \ldots ,g_{n - 1} } \right)$. To apply the results of the previous subsection, let $n^{ * } = 2^{m}$ be the smallest binary power greater than or equal to $n$, i.e., let $m = \left\lceil {\log_{2} n} \right\rceil$. If $n < n^{ * }$, set $g_{n} = g_{n + 1} = \cdots = g_{{n^{ * } - 1}} = e$, with the arrays $L_{k}^{ * }$ and $\bar{L}_{k}^{ * }$ of length $n_{k}^{ * }$ ($0 \le k < m$) as above. For general $n \ge 2$, consider the computation of the arrays $L_{k}$ ($0 < k < m$) in the upward phase of the pseudocode above.

We prove Proposition $P\left( k \right)$ (next) by induction on the level $0 \le k < m$.

#### **Proposition**$P\left( k \right)$

$L_{k} \left[ j \right] = L_{k}^{ * } \left[ j \right]$ for $0 \le j < n_{k}$, and $L_{k}^{ * } \left[ j \right] = e$ for $n_{k} \le j < n_{k}^{ * }$.

#### *Proof*

See Fig. 1. By construction, $P\left( 0 \right)$ holds. With $P\left( {k - 1} \right)$ in hand, the upward phase and Eq. (4) show that $L_{k} \left[ j \right] = L_{k}^{ * } \left[ j \right]$ for $0 \le j < \rho \left( {n_{k - 1} } \right)$. On one hand, if $n_{k - 1}$ is even, $\rho \left( {n_{k - 1} } \right) = n_{k}$, yielding $P\left( {k - 1} \right)$ immediately. On the other hand, if $n_{k - 1}$ is odd, $\rho \left( {n_{k - 1} } \right) = \left\lceil {n_{k - 1} /2} \right\rceil - 1 = n_{k} - 1$, so

8 $$\begin{aligned} L_{k} \left[ {n_{k} - 1} \right] & = L_{k} \left[ {\rho \left( {n_{k - 1} } \right)} \right] = L_{k - 1} \left[ {n_{k - 1} - 1} \right] = L_{k - 1}^{ * } \left[ {n_{k - 1} - 1} \right] \\ & = L_{k - 1}^{ * } \left[ {n_{k - 1} - 1} \right] \circ e = L_{k - 1}^{ * } \left[ {n_{k - 1} - 1} \right] \circ L_{k - 1}^{ * } \left[ {n_{k - 1} } \right] = L_{k}^{ * } \left[ {n_{k} - 1} \right], \\ \end{aligned}$$

the second equality reflecting the copy of the final element of $L_{k - 1}$ in the pseudocode; the third and fifth, $P\left( {k - 1} \right)$; and the sixth, Eq. (4). Equation (8) completes the proof that $L_{k} \left[ j \right] = L_{k}^{ * } \left[ j \right]$ for $0 \le j < n_{k}$. For the remaining indices $n_{k} \le j < n_{k}^{ * }$ of $L_{k}^{ * }$, note that $n_{k - 1} \le 2\left\lceil {n_{k - 1} /2} \right\rceil = 2n_{k} \le 2j < 2j + 1 < 2n_{k}^{ * } = n_{k - 1}^{ * }$. Then, $P\left( {k - 1} \right)$ and Eq. (4) show that $L_{k}^{ * } \left[ j \right] = L_{k - 1}^{ * } \left[ {2j} \right] \circ L_{k - 1}^{ * } \left[ {2j + 1} \right] = e \circ e = e$ for $n_{k} \le j < n_{k}^{ * }$. □

Note: $n_{m - 1} = 2 = n_{m - 1}^{ * }$, so $P\left( {m - 1} \right)$ shows that $L_{m - 1} = L_{m - 1}^{ * }$.

In the downward phase, the transposition function $\tau$ in Eq. (7) facilitates in-place computation for $\bar{L}_{k - 1}^{ * } \left[ j \right]$ in Eq. (7). Similarly, the minimization in the accessory index $\alpha_{k} \left( j \right) = \hbox{min} \left\{ {\tau \rho \left( j \right),n_{k} - 1} \right\}$ within $\bar{L}_{k}$ avoids storing a superfluous element $\bar{g}$ of $\bar{L}_{k}^{ * }$ within the penultimate element of any truncated complementary array $\bar{L}_{k}$ (see $\bar{L}_{1}^{ * } \left[ 3 \right]$, the dotted circle in Fig. 2).

We prove Proposition $\bar{P}\left( k \right)$ (next) by descending induction on the level $0 \le k < m$.

#### **Proposition**$\bar{P}\left( k \right)$

$\bar{L}_{k} \left[ j \right] = \bar{L}_{k}^{ * } \left[ j \right]$ for $0 \le j < n_{k}$, unless $n_{k}$ is odd and $j = n_{k} - 1$, in which case $\bar{L}_{k} \left[ {n_{k} - 1} \right] = \bar{L}_{k}^{ * } \left[ {n_{k} } \right]$.

#### *Proof*

The proposition $\bar{P}\left( {m - 1} \right)$ is true, because $\bar{L}_{m - 1} = L_{m - 1} = L_{m - 1}^{ * } = \bar{L}_{m - 1}^{ * }$, and $\bar{L}_{m - 1}$ has even length $n_{m - 1} = 2$. We now show that $\bar{P}\left( k \right)$ implies $\bar{P}\left( {k - 1} \right)$ for $0 < k < m$.

If $0 \le j < n_{k - 1}$, then $0 \le \rho \left( j \right) < \left\lceil {n_{k - 1} /2} \right\rceil = n_{k}$. For every $j$, either: (1) $n_{k}$ is odd and $\rho \left( j \right) = n_{k} - 1$; (2) $n_{k}$ is odd and $\rho \left( j \right) < n_{k} - 1$; or (3) $n_{k}$ is even. In Case 1, $\tau \rho \left( j \right) = n_{k}$ but $\alpha_{k} \left( j \right) = n_{k} - 1$. Because $n_{k}$ is odd, $\bar{P}\left( k \right)$ implies $\bar{L}_{k} \left[ {\alpha_{k} \left( j \right)} \right] = \bar{L}_{k} \left[ {n_{k} - 1} \right] = \bar{L}_{k}^{ * } \left[ {n_{k} } \right] = \bar{L}_{k}^{ * } \left[ {\tau \rho \left( j \right)} \right]$. In Case 2, $0 \le \alpha_{k} \left( j \right) = \tau \rho \left( j \right) < n_{k} - 1$, or in Case 3, $0 \le \alpha_{k} \left( j \right) = \tau \rho \left( j \right) < n_{k}$ and $n_{k}$ is even. In either case, $\bar{P}\left( k \right)$ implies $\bar{L}_{k} \left[ {\alpha_{k} \left( j \right)} \right] = \bar{L}_{k} \left[ {\tau \rho \left( j \right)} \right] = \bar{L}_{k}^{ * } \left[ {\tau \rho \left( j \right)} \right]$. Thus, regardless of whether Case 1, 2, or 3 pertains, $\bar{L}_{k} \left[ {\alpha_{k} \left( j \right)} \right] = \bar{L}_{k}^{ * } \left[ {\tau \rho \left( j \right)} \right]$ for every $0 \le j < n_{k - 1}$.

For $0 \le j < 2\rho \left( {n_{k - 1} } \right)$, the Jackknife Product algorithm in the downward phase and $P\left( {k - 1} \right)$ from the upward phase yield

9 $$\bar{L}_{k - 1} \left[ j \right] = L_{k - 1} \left[ j \right] \circ \bar{L}_{k} \left[ {\alpha_{k} \left( j \right)} \right] = \bar{L}_{k - 1}^{ * } \left[ j \right] \circ \bar{L}_{k}^{ * } \left[ {\tau \rho \left( j \right)} \right] = \bar{L}_{k - 1}^{ * } \left[ j \right]$$

if $j < n_{k - 1}$. On one hand, if $n_{k - 1}$ is even, $n_{k - 1} = 2\rho \left( {n_{k - 1} } \right)$, yielding $\bar{P}\left( {k - 1} \right)$ immediately. On the other hand, if $n_{k - 1}$ is odd, $n_{k - 1} = 2\rho \left( {n_{k - 1} } \right) + 1$, so $\bar{L}_{k - 1}$ has an additional, final element copied from $\bar{L}_{k}$:

10 $$\bar{L}_{k - 1} \left[ {n_{k - 1} - 1} \right] = \bar{L}_{k} \left[ {\alpha_{k} \left( {n_{k - 1} - 1} \right)} \right] = \bar{L}_{k}^{ * } \left[ {\tau \rho \left( {n_{k - 1} - 1} \right)} \right].$$

$P\left( {k - 1} \right)$ yields $L_{k - 1}^{ * } \left[ {n_{k - 1} } \right] = e$, so moreover,

11 $$\bar{L}_{k - 1}^{ * } \left[ {n_{k - 1} } \right] = L_{k - 1}^{ * } \left[ {n_{k - 1} } \right] \circ \bar{L}_{k}^{ * } \left[ {\tau \rho \left( {n_{k - 1} } \right)} \right] = \bar{L}_{k}^{ * } \left[ {\tau \rho \left( {n_{k - 1} } \right)} \right].$$

Because $n_{k - 1} - 1$ is even, $\rho \left( {n_{k - 1} - 1} \right) = \rho \left( {n_{k - 1} } \right)$. Equations (10) and (11) therefore yield $\bar{L}_{k - 1} \left[ {n_{k - 1} - 1} \right] = \bar{L}_{k - 1}^{ * } \left[ {n_{k - 1} } \right]$, so $\bar{P}\left( {k - 1} \right)$ holds. □

## Appendix B

This appendix gives combinatoric calculations for the circRNA-miRNA application in “Background” section. It therefore has some peripheral interest to this article.

### Motifs on a single circle

Consider $r$ points equally spaced around a circle (a “ring”). Call a set of $m$ consecutive points on the ring a “motif”. The following fixes $m$, so the notation can leave it implicit. Let $C_{r,k}$ count the ways of choosing $k$ non-overlapping motifs around the ring (i.e., the motifs have no point in common). Note: $C_{r,k} = 0$ if $r < mk$ or $k < 0$. Define the factorial function $n! = n\left( {n - 1} \right) \cdots 1$ and the binomial (combinatorial or Pascal) coefficient

12 $$\left( {\begin{array}{*{20}c} n \\ k \\ \end{array} } \right) = \frac{n!}{{k!\left( {n - k} \right)!}}$$

for $0 \le k \le n$ and 0 otherwise.

#### **Theorem**

Clearly, $C_{r,k} = 1$ when $r = mk$. For $r > mk$,

13 $$\begin{aligned} C_{r,k} &= \left( {\begin{array}{*{20}c} {r - 1 - \left( {m - 1} \right)k} \\ k \\ \end{array} } \right) + m\left( {\begin{array}{*{20}c} {r - m - \left( {m - 1} \right)\left( {k - 1} \right)} \\ {k - 1} \\ \end{array} } \right) \\ &= \left( {\begin{array}{*{20}c} {r - \left( {m - 1} \right)k - 1} \\ k \\ \end{array} } \right) + m\left( {\begin{array}{*{20}c} {r - \left( {m - 1} \right)k - 1} \\ {k - 1} \\ \end{array} } \right) \\ \end{aligned}$$

counts the ways of placing $k$ motifs around the ring. For convenience below and for consistency with Eq. (13), $C_{r,0} = 1$ for $r \ge 0$.

#### *Proof*

Consider a line segment containing $r$ equally spaced points, and let $L_{r,k}$ count the ways of choosing $k$ non-overlapping motifs, each of $m$ consecutive points, on it. First, $C_{r,k} = L_{r - 1,k} + mL_{r - m,k - 1}$, proved as follows. Number the ring points arbitrarily as positions $1,2, \ldots ,r$, and place the $k$ motifs as follows. Consider position 1, which might have no motif. If so, place the $k$ motifs in a line consisting of $r - 1$ positions $2,3, \ldots ,r$ ($L_{r - 1,k}$ ways). Otherwise, place the first motif in one of the $m$ positions in which it covers position 1, and then place the remaining $k - 1$ motifs in a line consisting of $r - m$ positions ($mL_{r - m,k - 1}$ ways). Each such configuration corresponds to placing $k$ leftmost end-positions on the line. For each of the $k$ motifs, delete $m - 1$ positions on the ring, all but its leftmost end-position. Each of the configurations for $k$ motifs therefore corresponds to choosing $k$ positions from the $r - \left( {m - 1} \right)k$ positions remaining, yielding the remaining factors in Eq. (13).

### Motifs on several circles

Now, let $C_{r\left( 1 \right),r\left( 2 \right), \ldots ,r\left( N \right);K}$ count the ways of distributing $K$ non-overlapping motifs (all of $m$ consecutive points) around several rings, the rings’ points numbering $r\left( n \right)$ ($n = 1,2 \ldots ,N$). Without loss of generality, assume $r\left( n \right) \ge m$ ($n = 1,2 \ldots ,N$). (Otherwise, discard all rings with $r\left( n \right) < m$.) The following recursion holds: $C_{r\left( 1 \right),r\left( 2 \right), \ldots ,r\left( N \right);K} = 0$ for $K < 0$ or $K > \sum\nolimits_{n = 1}^{N} {\left\lfloor {r\left( n \right)/m} \right\rfloor }$, $C_{r\left( 1 \right),r\left( 2 \right), \ldots ,r\left( N \right);0} = 1$, and otherwise

14 $$C_{r\left( 1 \right),r\left( 2 \right), \ldots ,r\left( N \right);K} = \sum {C_{r\left( N \right),k} C_{{r\left( 1 \right),r\left( 2 \right), \ldots ,r\left( {N - 1} \right);K - k}} } ,$$

where on the right, the index of summation $k$ runs from $\hbox{max} \left\{ {0,K - \sum\nolimits_{n = 1}^{N - 1} {\left\lfloor {r\left( n \right)/m} \right\rfloor } } \right\}$ up to $\hbox{min} \left\{ {K,\left\lfloor {r\left( N \right)/m} \right\rfloor } \right\}$. Equation (13) initializes the convolution recursion in Eq. (14) with $C_{r\left( 1 \right),K}$.

### Relation to the Jackknife Product algorithm

To apply the Jackknife Product algorithm in the circRNA–miRNA application, let $g_{i} = \left( {C_{r\left( i \right),0} ,C_{r\left( i \right),1} , \ldots ,C_{r\left( i \right),K} } \right)$ ($i = 1, \ldots ,N$) in the (commutative) semigroup $\left( {G, \circ } \right)$ of non-negative integer vectors with indices $k = 0,1, \ldots ,K$, under the convolution operation.
